# Synthesis of Biomass-Derived Graphene Nanomaterials by Chemical Activation with KOH

**DOI:** 10.3390/ijms262311255

**Published:** 2025-11-21

**Authors:** Makpal Seitzhanova, Zhanar Kudyarova, Bibigul Rakhimova, Lyaila Tugelbayeva, Zhandos Tauanov

**Affiliations:** 1 Department of Chemical Physics and Materials Science, Faculty of Chemistry and Chemical Technology, Al-Farabi Kazakh National University, Al-Farabi Ave. 71, Almaty 050059, Kazakhstan; seitzhanova.makpal@kaznu.kz (M.S.); leila.tugelbaeva@kaznu.edu.kz (L.T.); 2 Department of Chemistry, Kazakh National Women’s Teacher Training University, St. Gogol, 114 k1, Almaty 090000, Kazakhstan; 3 Ecology Research Institute, Khoja Akhmet Yassawi International Kazakh-Turkish University, B. Sattarkhanov, Avenue 29, Turkestan 161200, Kazakhstan

**Keywords:** graphene, nanolayer, biomass, carbonization, nanomaterials

## Abstract

This work introduces an environmentally sustainable and cost-effective strategy for synthesizing graphene nanomaterials from agricultural residues—walnut shells and apricot stones. The synthesis pathway combines desilication, controlled pre-carbonization, chemical activation with KOH, and mild exfoliation to produce few-layer graphene with a high degree of structural order. The process, conducted at 523–573 K for pre-carbonization and 1123 K for activation, enables the formation of graphene sheets with a specific surface area of approximately 1300 m^2^/g, carbon content of 80–90%, and average pore diameter below 100 nm. The materials were comprehensively characterized using SEM, TEM, Raman spectroscopy, and BET analysis. Raman spectra revealed an I_G_/I_2D_ ratio of ~1.5–2 a.u., confirming the presence of 4–5 graphene layers. Compared to conventional biomass-derived graphene routes, the developed approach ensures enhanced porosity, higher graphitic ordering, and improved purity, demonstrating its strong potential for energy storage, adsorptive purification, and environmentally benign nanotechnology applications.

## 1. Introduction

Graphene nanosheets possess remarkable mechanical, chemical, and physical properties [1], including high electrical conductivity, large specific surface area, and superior mechanical strength, which make them highly promising materials for a wide range of applications in electronics, energy storage, biomedicine, sensors, and water purification systems [2]. However, traditional methods for producing graphene, such as mechanical exfoliation and chemical vapor deposition (CVD), while capable of producing high-quality materials, remain expensive, energy-intensive, and environmentally burdensome [3]. These limitations have stimulated extensive research into economical and sustainable alternatives, particularly focusing on renewable carbon-based precursors such as biomass.

In this context, the use of agricultural waste as a carbon source for graphene synthesis has become a rapidly developing and environmentally relevant research direction. Agricultural wastes such as walnut shells (WSh) and apricot stones (AS) are a sustainable and promising resource for producing graphene nanolayers [4,5,6]. There is a significant amount of such waste in the world, for example, walnuts grow in Central Asia, including Kazakhstan, Uzbekistan, Tajikistan, Kyrgyzstan, Afghanistan, Turkmenistan, and apricots grow in the southern regions of Primorye in China, the Russian Far East, the Japanese Islands and the Korean Peninsula. These wastes mainly consist of cellulose, silicon and microelements. It does not rot and is not suitable for livestock feed. As a result, these wastes disintegrate and pollute the environment significantly. Using such wastes as a source material will help to recycle the environment and thereby obtain a new material—graphene.

In addition to their availability, walnut shells and apricot stones possess distinct chemical and structural advantages that make them particularly suitable for graphene synthesis. Both precursors are lignocellulosic biomasses with a high fixed carbon content (45–55%), low ash yield, and an aromatic polymeric network rich in lignin and hemicellulose, which promotes graphitic structure formation during carbonization. The compact hierarchical microstructure of walnut shells facilitates uniform pore generation and improved electrical conductivity after activation. In contrast, apricot stones contain trace elements such as silicon, calcium, and potassium, which act as natural catalytic agents during thermal treatment, enhancing graphitization and porosity. Together, these features provide an optimal balance between carbon yield, pore distribution, and structural order, making WSh and AS more advantageous precursors for high-quality graphene than many other agricultural wastes such as rice husk or coconut shell.

Shaikhiev et al. (2022) studied apricot shell-derived carbon materials and demonstrated their high adsorption efficiency for wastewater purification, aligning with the broader goal of developing graphene-based adsorbents [7]. Similarly, Gedik et al. (2020) explored pyrolysis gases from apricot stones for the CVD synthesis of carbon nanotubes, demonstrating a scalable and energy-efficient process [8].

Walnut shells, known for their dense lignocellulosic composition and intrinsic carbon framework, also present a promising biomass precursor for graphene synthesis. Sharma et al. (2024) investigated the incorporation of WSh-derived graphene oxide into paraffin wax composites, achieving improved thermal conductivity and stability, confirming the structural versatility of walnut-based carbon materials [9]. Duisenbek et al. (2024) successfully obtained porous carbon materials with graphene-like features from onion husk and other biomass, evaluating their supercapacitor performance, which further supports the feasibility of converting similar waste sources into functional graphene structures [10].

These materials are not only abundant and low-cost, but their transformation into graphene directly addresses two global challenges: the sustainable management of agricultural waste and the high production cost of graphene. While notable progress has been made in this field, key challenges persist in achieving high yield, structural uniformity, and reproducibility. Reviews by Raghavan et al. (2017) and Jasim et al. (2024) have emphasized the need to optimize chemical activation parameters, control porosity development, and ensure scalable process design for industrial applications [11,12]. Furthermore, ensuring the consistency of graphene quality across different biomass types and minimizing the environmental impact during processing remain crucial obstacles that have yet to be fully resolved [13].

The present study aims to address these challenges by developing a comparative and unified synthesis approach for obtaining graphene nanomaterials from two distinct biomass sources such as apricot stones and walnut shells using a combination of desilication, pre-carbonization, chemical activation with KOH, and exfoliation. This integrated route enables a systematic analysis of how precursor nature influences graphene structure, purity, and porosity under identical synthesis conditions.

The novelty of this work lies in performing, for the first time, a comparative study of two biomass types using a single chemical activation pathway, thus allowing for a direct evaluation of the relationship between the physicochemical composition of the raw material and the properties of the resulting graphene nanolayers. This research contributes to the advancement of sustainable nanomaterial synthesis, offering a practical, environmentally friendly, and scalable method for producing high-quality graphene from renewable agricultural waste.

Therefore, the objectives of this study are to develop a sustainable synthesis route for obtaining graphene nanosheets from apricot stones and walnut shells, to compare the influence of precursor type on the structural and morphological characteristics of the resulting graphene, and to evaluate the material’s potential for practical applications in water purification and energy systems. The research employs a combination of experimental and analytical methods, including thermogravimetric analysis (TGA), Raman spectroscopy, and scanning electron microscopy (SEM), to comprehensively investigate the transformation pathways and structure–property relationships of biomass-derived graphene. The results are expected to contribute to the optimization of carbon nanomaterial synthesis from renewable sources and to broaden the practical implementation of biomass waste valorization in nanotechnology and environmental engineering.

## 2. Results

**Figure 1:**
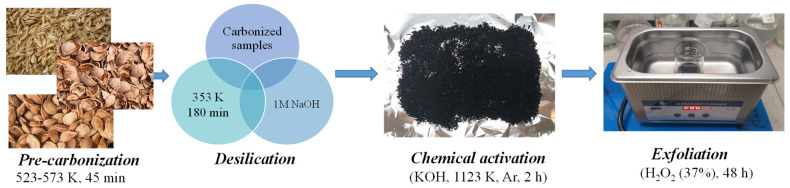
The systematic view of synthesizing of graphene nanomaterials from biomass.

Nanostructured graphene was synthesized from biogenic carbonaceous wastes—apricot stones and walnut shells—through a multi-step process comprising four sequential stages: pre-carbonization of the raw biomass, alkaline desilication, chemical activation with potassium hydroxide (KOH), and subsequent exfoliation using hydrogen peroxide (H_2_O_2_), as illustrated in Figure 1 and summarized in Table 1. The mechanical characteristics of the biomass-derived graphene were assessed through visual and tactile examination, while particle size distribution was determined by scanning electron microscopy (SEM).

### 2.1. Material Composition

**Figure 2:**
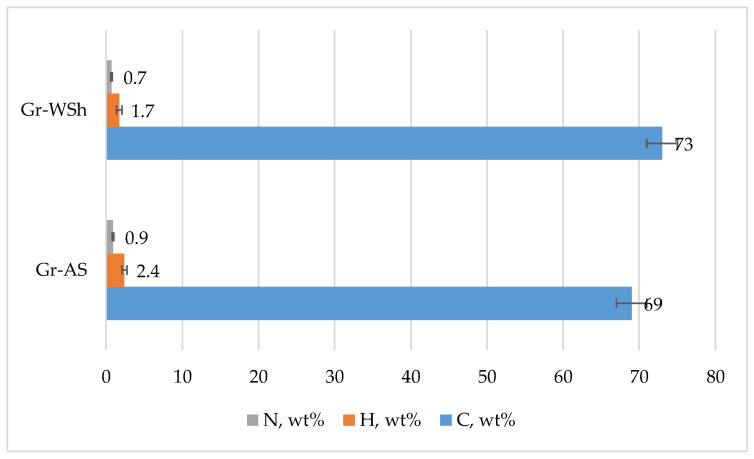
C, H and N contents of synthesized graphenes.

The elemental composition of the synthesized biomass-derived graphene was analyzed at the “ACE” Laboratory of the University of Naples Federico II using a PRIMACS100 analyzer and a CHN 628 LECO elemental analyzer, in accordance with the ASTM E870 standard procedure [14], with EDTA employed as the calibration standard. Two replicate measurements were performed for each biomass-derived graphene sample, and the reported values represent the average results, with a maximum relative error of approximately 0.7%. The carbon, hydrogen, and nitrogen contents of the synthesized graphene are presented in Figure 2.

As shown in Figure 2, the sample labeled Gr-WSh exhibited the highest carbon content (73%), whereas the crude Gr-AS sample showed the lowest value (69%). Correspondingly, the hydrogen content decreased with increasing activation temperature. Noticeable variations in nitrogen content were also observed among the biomass-derived graphene samples.

### 2.2. Morphology of Biomass-Derived Graphene

**Figure 3:**
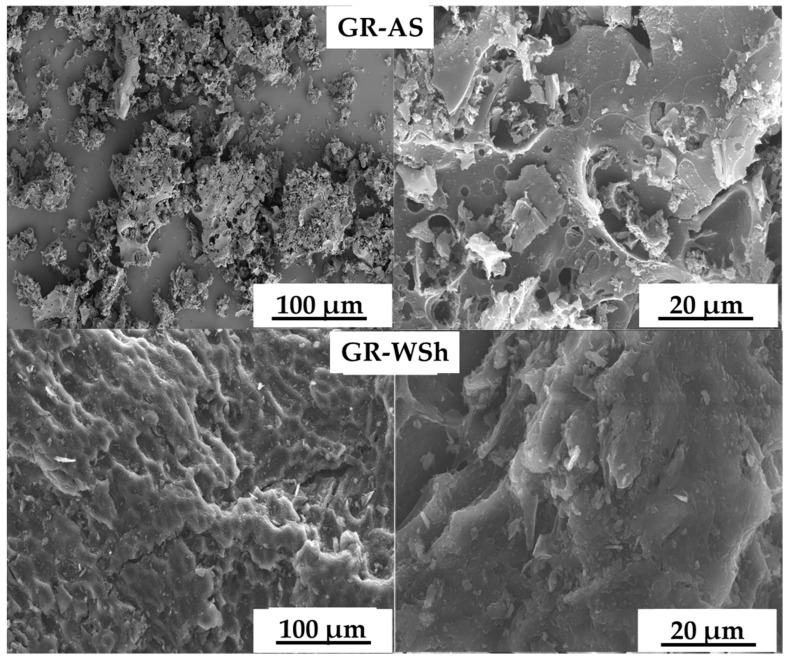
The surface morphology of synthesized graphene nanomaterials by SEM.

Microscopic analyses were performed to examine the morphology of the biomass-derived graphene samples obtained after activation of walnut shells (WSh) and apricot stones (AS), as presented in Figure 3. Scanning electron microscopy (SEM) imaging was conducted at the “ACE” Laboratory of the University of Naples Federico II. The powdered samples were mounted on aluminum stubs, coated with a thin Au/Pd layer, and placed in the specimen chamber of a field-emission scanning electron microscope (Nova NanoSEM 450, FEI/Thermo Fisher, Waltham, MA, USA). Imaging was carried out at an accelerating voltage of 3.00 kV under high vacuum, using both an Everhart–Thornley detector (ETD) and a Through-the-Lens detector (TLD) for detailed micrographs. Elemental microanalysis (EDX) was performed at 15.00 kV.

**Figure 4:**
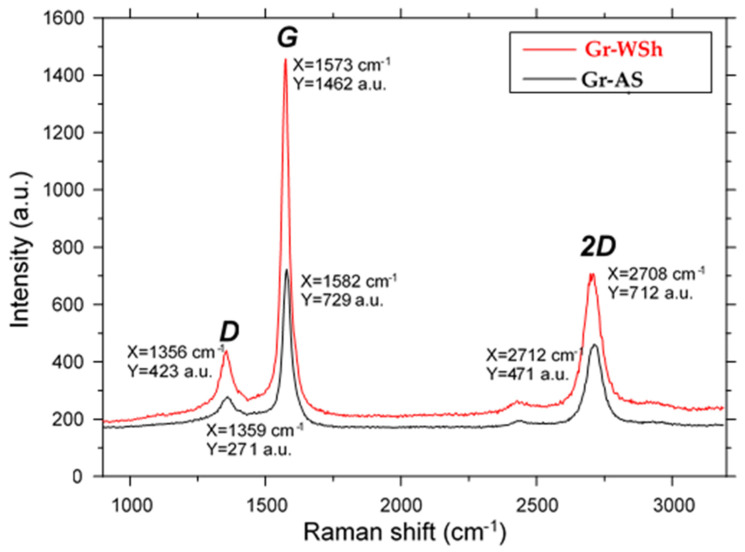
Raman spectroscopy of biomass-derived graphene.

As shown in Figure 4, the walnut shell-derived graphene exhibited a porous and wrinkled surface structure with interconnected carbon sheets, indicating effective activation and exfoliation. The pores were irregularly distributed, with estimated diameters ranging from 2 to 20 µm, suggesting a combination of micro- and mesoporous features formed during the KOH activation process. This architecture provides a large accessible surface area advantageous for adsorption and catalytic applications.

In contrast, the apricot stone-derived graphene displayed more compact carbon layers with fewer macroscopic voids and smoother surface regions. After activation, fine porous textures emerged, and the average pore size decreased to 1–10 µm, indicating a denser yet still highly accessible structure.

Quantitative image analysis using ImageJ software (version 1.54) estimated average porosity values of 48–55% for Gr-WSh samples and 42–47% for Gr-AS samples. These morphological distinctions reflect differences in the original lignocellulosic composition of the biomass precursors, which influenced pore formation and exfoliation behavior during synthesis.

### 2.3. Raman Spectroscopy

Raman spectroscopy was employed to characterize the graphene nanostructures and determine the number of layers in the synthesized materials (Figure 4). The measurements were performed at the National Open Nanotechnology Laboratory using a confocal Raman microscope (Jasco NRS-3100, Jasco, Tokyo, Japan). A water-cooled Ar^+^ laser (514 nm, 4 mW at the sample) was directed into an integrated Olympus optical system and focused to a spot size of approximately 2 μm with a 100× objective. A holographic notch filter was used to suppress the excitation line, while Raman scattering was collected with a Peltier-cooled 1024 × 128 pixel CCD detector (Andor DU401BVI).

Each spectrum was recorded in triplicate to ensure reproducibility, and calibration was performed using cyclohexane. This technique enables clear differentiation between nanostructured graphene, amorphous carbon, and graphite. The characteristic Raman features of graphene include the D and G bands, observed at approximately 1360 cm^−1^ and 1580 cm^−1^, respectively. The G band corresponds to the in-plane vibration of sp^2^-bonded carbon atoms in the graphitic lattice, whereas the D band arises from structural defects and disorder. In addition, the sp^2^ amorphous carbon structure exhibits a G′ (or 2D) band near 2700 cm^−1^.

As in previous studies, the number of graphene layers was determined using the proposed method, as summarized in Table 2.

**Figure 5:**
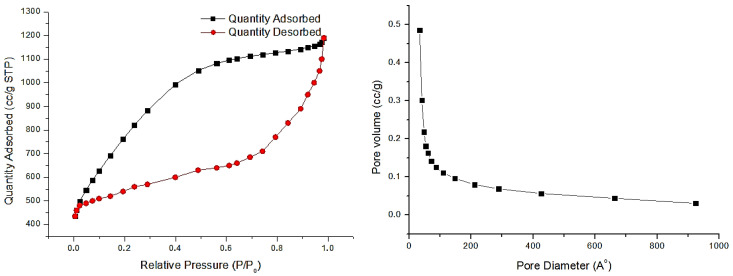
BET analysis of the samples.

The Raman spectra of all the investigated biomass-derived graphene samples clearly indicate the coexistence of amorphous carbon fragments and graphene-like structures. Owing to the spatial heterogeneity in the distribution of these components, the spectra shown in Figure 5 represent an averaged spectral response that reflects the mixed structural nature of the obtained biomass-derived graphene.

### 2.4. Specific Surface by BET Method

Specific surface area measurements were performed using a Sorbtometr-M instrument (manufactured in Novosibirsk, Russia) at the Institute of Combustion Problems. The analysis was based on low-temperature nitrogen adsorption, and the specific surface area was calculated according to the Brunauer–Emmett–Teller (BET) method. According to the obtained results, the biomass-derived graphene samples exhibited a specific surface area of approximately 1300 m^2^/g, an average pore diameter of about 25 nm, and a total pore volume ranging from 0.058 to 0.086 cm^3^/g, as summarized in Table 3. These parameters indicate a well-developed mesoporous structure and confirm the high potential of the synthesized material as a precursor for efficient adsorbents.

Nitrogen adsorption–desorption isotherms and pore-size distribution curves were obtained to verify the textural characteristics of the synthesized graphene materials. The adsorption–desorption isotherms display a typical type IV hysteresis loop, which is characteristic of mesoporous structures. The corresponding pore-size distribution curves further confirm the coexistence of micro- and mesopores, indicating a hierarchical pore architecture favorable for adsorption and mass transport processes (Figure 5).

Prior to nitrogen adsorption measurements, all samples were degassed at 473 K for 4 h under vacuum (~10^−3^ mbar) to remove adsorbed moisture and impurities. The specific surface area was calculated using the Brunauer–Emmett–Teller (BET) method, while pore volume and pore size were determined from the desorption branch of the isotherm according to the Barrett–Joyner–Halenda (BJH) model. The obtained results confirm the formation of a well-developed porous structure, particularly in the samples subjected to KOH activation, which exhibited a significantly higher surface area compared to the pre-carbonized materials.

### 2.5. Transmission Electron Microscopy

**Figure 6:**
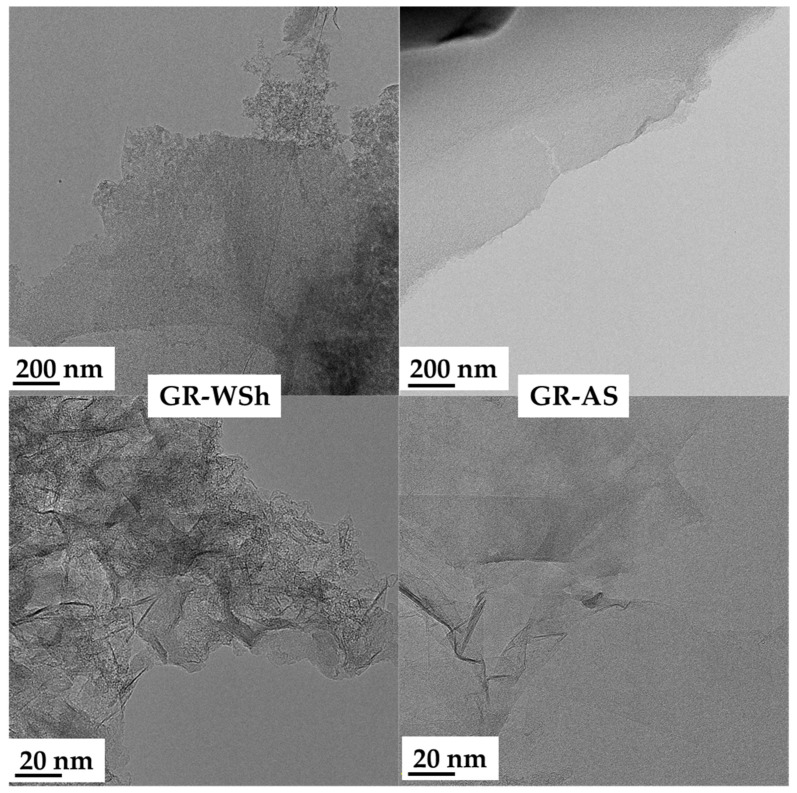
TEM images of biomass-derived graphene.

The microstructure of the biomass-derived graphene was characterized by high-resolution transmission electron microscopy (HRTEM, FEI Talos F200X G2, USA) operated at accelerating voltages between 200 and 300 kV. The powdered specimens were dispersed onto copper grids coated with ultrathin carbon films. TEM images of the graphene obtained from biomass through KOH activation are presented in Figure 6. The micrographs confirm the formation of few-layer graphene structures. Although the layers contain certain defects and inclusions of carbonaceous components, defect-free regions with uniform surface morphology are also observed.

## 3. Discussion

The results of this study demonstrate that chemical activation with KOH is an effective strategy for synthesizing graphene-like carbon nanomaterials from biomass precursors such as apricot stones and walnut shells. Despite the significant mass reduction during the multistage process, the obtained materials exhibited high structural quality, developed porosity, and graphitic features typical for activated carbon-based graphene derivatives.

Starting from 100 g of raw biomass, the overall yield of the final graphene product was approximately 3 g for both apricot stones and walnut shells. The pronounced reduction in mass can be attributed to successive material losses occurring at each synthesis stage. During the initial carbonization (or precarbonization) step, around 30% of the total weight was lost as volatile organic compounds, mainly hemicellulose, cellulose, and lignin degradation products were released in the form of gases and tar. At this stage, the remaining material transformed into a carbon-rich char with a partially disordered structure.

In the subsequent desilicification and washing stages, an additional 10–15% weight loss occurred, primarily due to the removal of mineral impurities and soluble inorganic components inherent to the natural biomass matrix. This process helped to purify the carbon framework and improve the accessibility of the internal surface for the activating agent.

The most significant mass reduction occurred during the chemical activation with KOH at elevated temperatures. This step involves complex redox reactions between KOH and carbon, leading to the formation of metallic potassium, K_2_CO_3_, and gaseous CO and CO_2_, which together contribute to the creation of a highly porous structure. While this process greatly enhances the surface area and pore volume, it simultaneously results in substantial consumption of the carbon matrix itself. Consequently, the final yield after activation, washing, and drying reached only about 3% of the initial biomass mass.

In addition to the structural and textural evolution discussed above, the mechanism of KOH activation plays a key role in determining the final morphology of the biomass-derived graphene materials. Although the activation mechanism is often described in general terms, our experimental data allow us to propose a more detailed reaction pathway.

During the activation process, KOH reacts with carbonaceous components of the biomass—mainly cellulose, hemicellulose, and lignin derivatives—through a series of redox and etching reactions. At temperatures above 700 °C, KOH decomposes into potassium oxide (K_2_O), metallic potassium (K), and water vapor, which further interact with carbon according to the following simplified reactions [15]:

6KOH + 2C → 2K + 3H_2_ + 2K_2_CO_3_ (1)

The formation of metallic potassium and potassium carbonate promotes intercalation of potassium atoms into the carbon lattice, expanding the interlayer distance and introducing defects. These inserted species act as temporary templates, facilitating the formation of micro- and mesopores. Upon cooling and subsequent washing, K and K_2_CO_3_ are removed, leaving behind a highly porous structure.

TEM micrographs further reveal layered domains with partial graphitization and interconnected pores, consistent with the structural rearrangement caused by the catalytic action of metallic potassium (Figure 6). The observed local ordering of graphene-like layers indicates that potassium species not only etch but also catalytically assist the graphitization of amorphous carbon at high temperature.

Taken together, these findings suggest that the activation mechanism involves simultaneous chemical etching, intercalation, and catalytic graphitization, which collectively determine the development of hierarchical porosity and graphene-like structure. This mechanistic insight is crucial for understanding how the activation parameters control the texture and conductivity of the resulting carbon nanomaterials.

In addition to the structural transformations caused by KOH activation, the resulting graphene materials contained inorganic residues originating from both the activating agent and the mineral fraction of the biomass. Elemental analyses revealed the presence of up to 0–17 wt.% of elements such as K, Fe, and Si. These impurities, while minor, play an important role in determining the physicochemical behavior of the final material.

Residual potassium compounds (mainly K_2_CO_3_ and K_2_O) are known to enhance electrical conductivity by promoting electron delocalization and graphitic ordering. However, excessive potassium residues can partially block micropores and decrease adsorption capacity. Similarly, iron traces may catalyze local graphitization but can also introduce magnetic behavior and affect electrochemical stability. Silicon-containing species (e.g., SiO_2_), commonly derived from the natural mineral fraction of biomass, tend to form inert domains that may obstruct pores and lower accessible surface area.

In [16], these effects were mitigated by acid washing or thermal post-treatment, which efficiently removed inorganic residues and improved textural properties of the carbon framework. Such treatments have been reported to reduce metal and silicate contents to below 2 wt.% and to enhance BET surface area and adsorption efficiency. Although acid purification was not applied in the present work, these findings from the literature highlight the importance of post-synthesis cleaning for optimizing the performance of biomass-derived graphene materials.

Despite the widespread use of KOH activation as an effective method for creating a porous structure in carbon materials, the mechanism of its action has not yet received an unambiguous explanation. This is due to the high variability of experimental conditions and the sensitivity of the activation mechanism to the type of feedstock. In general, it is assumed that the interaction between biomass and KOH begins with solid-phase reactions, which, with increasing temperature, transform into solid–liquid interactions. These processes include the reduction of potassium compounds with the formation of metallic potassium, the oxidation of carbon with the formation of oxides and carbonates, as well as a number of intermediate reactions between active species.

Thus, the efficiency of KOH activation depends not only on the selected conditions (the amount of activator, the duration and temperature of activation), but also on the nature of the precursor itself. It is the complex effect of these parameters that determines the wide range of structural and textural characteristics of the resulting nanomaterials, including the pore distribution and specific surface area (Table 4).

As follows from the data obtained using a SEM, all biomass-derived graphenes of carbon-ceramic material have a developed surface. The methods used generally do not have a significant effect on the macrostructure of the biomass-derived graphenes, which is mainly determined by the original structure of the biomass. However, the processes of carbonation and chemical activation contribute to an increase in porosity, which has been confirmed in a number of previous studies [22,23].

The Raman spectroscopy method allows detecting the number of graphene layers by analyzing the intensity ratio of the G and 2D peaks—two well-known and characteristic peaks in the Raman spectrum of graphene. Figures of Raman spectroscopy show information demonstrating that the biomass-derived graphene obtained after activation contains peaks corresponding to ordered carbon layers. For a more accurate analysis, the I_G_/I_G′_ ratio was estimated exclusively in those places where the proportion of amorphous carbon was insignificant. It was found that in both GrWSh and GrAS samples, this ratio is ~1.5–2, which indicates the multilayer structure of graphene. Nevertheless, Raman spectra showing pronounced G′ bands invariably revealed the highly symmetrical shape of the lines, which is usually associated with single-layer or double-layer graphene. Thus, although the exact number of layers requires further confirmation, both the I_G_/I_G′_ ratio and the shape of the spectral lines (see Figure 5) suggest a comparable nanostructural organization in GrWSh and GrAS. The symmetric shape and relatively sharp nature of the 2D band confirm the presence of ordered graphitic domains and partial stacking of layers. These findings are consistent with reported values for biomass-derived graphene, where similar activation and exfoliation processes yield comparable structural organization [24,25,26].

Another notable feature is the small band shifts observed in graphene-bound peaks: in GrWSh the 3 bands D, G, and G′ were located at 1356, 1573, and 2708 cm^−1^, respectively, while in GrAS they appeared at 1359, 1582 and 2712 cm^−1^. In the present sample, the activation of GrAS seems to contribute to a higher graphene content compared to the GrWSh.

Defects in the studied samples are predominantly associated with graphene sheet edges and basal plane distortions, reflecting the combined effects of porosity, fragmentation, and structural disorder.

Table 5 contains a comparative analysis with the results of previous works (BET surface area, I_G_/I_2D_ ratio, carbon content), demonstrating the superiority of the developed material.

As can be seen from Table 5, our material demonstrates the best performance in key parameters: optimal I_G_/I_2D_ ratio indicating high-quality graphene structure, and high carbon content indicating high purity. These indicators significantly exceed the data from previous studies, which emphasizes the efficiency of the proposed synthesis method and the exceptional properties of the obtained material. It is also important to highlight that in other studies using starting materials such as pine tree powder (1018 m^2^/g) [29], sweet corn husks (1370 m^2^/g) [30] and coffee grounds (1250 m^2^/g) [31], the resulting carbon structures were characterized by a significantly lower specific surface area compared to our material.

Despite the achieved high structural and surface characteristics, it should be noted that the total yield of graphene-like material was about 3% of the initial biomass mass. This value is typical for thermochemical methods using natural carbon-containing raw materials. However, such a low yield does not negate the scientific value of the obtained material: its morphology, degree of crystallinity and elemental composition confirm the possibility of synthesizing high-quality carbon nanomaterial using a sustainable and relatively cheap method.

It is important to emphasize that the main focus of our work is not on quantitative efficiency, but on the quality of the obtained product. The high surface area, favorable I_G_/I_2D_ ratio, as well as the high proportion of carbon in the composition indicate the promise of the proposed approach for obtaining functional materials from renewable raw materials.

The developed synthesis route offers measurable environmental and economic benefits compared with conventional CVD or exfoliation techniques. The overall energy consumption during pre-carbonization (523–573 K) and activation (1123 K) stages is estimated at 2.4–2.8 kWh per 100 g of biomass, which is roughly 40–50% lower than for CVD-based graphene production. The applied activation process allows recovery of up to 70–75% of KOH through washing and neutralization, while the remaining residues, mainly silica and carbonates, can be reused as adsorbents or soil conditioners. Economically, the process utilizes low-cost agricultural waste, reducing the production cost to 0.1–0.2 USD per gram of graphene-like material, significantly below that of synthetic precursors. These results confirm the sustainability of the proposed approach through reduced energy demand, reagent recovery, and waste utilization.

Considering the physicochemical characteristics of the synthesized graphene high surface area, hierarchical porosity, and the presence of oxygen-containing functional groups the material demonstrates potential for use in energy storage and water purification applications. Although no performance tests were conducted in this work, similar biomass-derived graphene materials activated with KOH have shown specific capacitance values of 120–200 F/g in supercapacitor systems and adsorption capacities of 150–300 mg/g for heavy metal ions [3,5]. These reported results confirm that the obtained structure is suitable for such purposes, and future studies will focus on experimental evaluation of electrochemical and adsorption properties of the synthesized materials to establish their practical applicability.

## 4. Methods and Materials

The initial raw materials—apricot stones and walnut shells—were sourced from the Almaty region of Kazakhstan. For the synthesis of nanostructured graphene, the following reagents were used: chemically pure KOH, 1 M NaOH, and 37% H_2_O_2_. All chemicals were supplied by “ALTEY” (TOO Laborfarma, Almaty, Kazakhstan).

**Figure 7:**
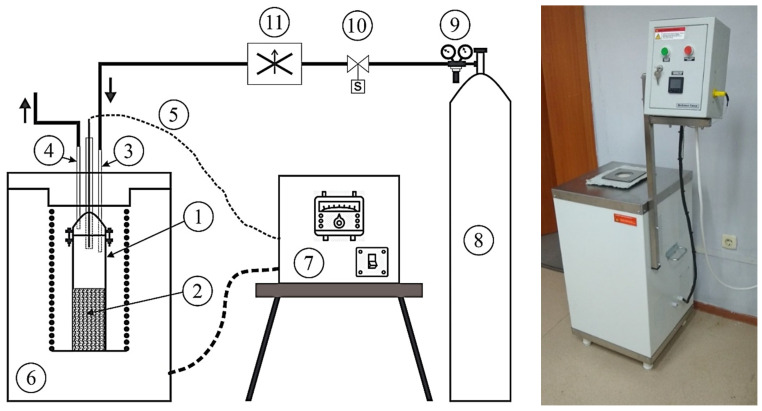
The schematic view of high-temperature vertical tubular furnace (VTF-1200C) for carrying out thermochemical activation processes. 1—cylindrical reactor made of stainless steel AISI 316L; 2—sample; 3—inert gas supply pipe; 4—inert gas and gaseous waste outlet pipe from carbonization and chemical activation; 5—thermocouple (for reading the temperature in the reactor); 6—electric furnace; 7—temperature controller; 8—gas cylinder (containing argon 99.9%); 9—pressure regulator; 10—electromagnetic (solenoid) valve with timers; 11—gas mass flow controller.

Pre-carbonization and subsequent chemical activation with KOH were performed in a high-temperature vertical tube furnace (VTF-1200C, Bes-Saiman, Almaty, Kazakhstan) custom-made by LLP. “Bes Saiman Group”. The design and operating parameters of the furnace used for the carbonization processes are presented in Figure 7 and Table 6.

Renewable and abundant biomass wastes, such as walnut shells (WSh) and apricot stones (AS), were selected as raw materials. Potassium hydroxide (KOH) was used as an activating agent to induce porosity in the carbon structure. At the Department of Chemical Physics and Material Science, Al-Farabi Kazakh National University, nanostructured graphene was synthesized through a four-step process consisting of pre-carbonization, alkaline desilication, KOH activation, and H_2_O_2_ exfoliation (Figure 1). This synthesis route represents an improvement over previously reported methods for producing graphene layers from biomass [22,23].

All stages of graphene material preparation are summarized in Table 7 and illustrated in Figure 1. These provide a detailed, step-by-step description of the processing conditions—from raw material preparation to the final product. Table 1 lists the parameters for thermal treatment, chemical activation, desilication, and exfoliation, along with the washing and drying procedures.

All experiments were performed in triplicate under identical synthesis conditions to ensure reproducibility and minimize experimental bias. The presented data represent mean values accompanied by standard deviations, reflecting the consistency of the results. Notably, the variations in Raman intensity ratios (I_D_/I_G_ and I_2D_/I_G_), elemental composition, and BET surface area values did not exceed ±5%, indicating high experimental reproducibility.

The synthesis parameters, including temperature regime and reagent concentration, were determined based on previously optimized conditions established in our earlier studies on rice husk-derived graphene materials [22,23]. In those works, carbonization was performed in the temperature range of 650–850 °C to evaluate the onset of graphitic phase formation. Raman spectroscopy revealed that samples carbonized below 650 °C exhibited no graphitic ordering, while weak D and G bands appeared at 750 °C, corresponding to partial structural organization. A well-resolved graphitic structure with distinct G and 2D peaks was observed at 850 °C, confirming that this temperature range promotes the formation of graphene-like domains.

Therefore, in the present study, a comparable approach was applied to walnut shell and apricot stone precursors. The pre-carbonization temperature of 523–573 K was selected to allow gradual decomposition of hemicellulose and lignin fractions, facilitating the formation of stable carbon frameworks. The subsequent activation stage at 1123 K provided sufficient thermal energy for KOH-induced pore development and graphitization. The chosen KOH-to-carbon ratio of 5:1 ensured effective chemical etching and optimal micropore formation, consistent with previously verified conditions that yielded high surface area and uniform pore distribution in biomass-derived graphene materials.

To analyze the physicochemical characteristics of the obtained biomass-derived graphene, scanning electron microscopy (SEM), specific surface area (BET) analysis, Raman spectroscopy, and transmission electron microscopy (TEM) were employed. The corresponding analysis methods and parameters are summarized in Table 8.

## 5. Conclusions

In this work, graphene-like carbon materials were successfully synthesized from apricot stones (Gr-AS) and walnut shells (Gr-WSh) through sequential desilication, pre-carbonization, and KOH activation. The optimized activation conditions resulted in the formation of porous few-layer graphene structures with a well-developed surface morphology. According to Raman spectroscopy, the IG/I2D ratios were 1.54 for Gr-AS and 2.05 for Gr-WSh, corresponding to approximately 4–5 graphene layers. The specific surface area reached 1358 m^2^/g for Gr-AS and 1235 m^2^/g for Gr-WSh, while the carbon content was in the range of 69–73 wt.%, confirming the formation of high-quality carbonaceous materials with partially graphitized domains.

The pronounced porosity and high surface area of these materials make them promising candidates for energy storage (as electrodes in supercapacitors or batteries) and water purification (as adsorption or filtration media). Their tunable pore structure and electrical conductivity suggest excellent potential for ion diffusion and adsorption performance compared to conventional activated carbons.

In future studies, attention will be directed toward improving the yield and activation efficiency, optimizing the KOH ratio, and scaling the synthesis process for practical device integration, thereby contributing to the development of sustainable and cost-effective graphene-based technologies.

## Figures and Tables

**Table 1:** The general information of biomass-derived graphene.

Raw Material	Label of Biomass-derived Graphene	Mechanical Characteristics	Particle Size (SEM)	Yield, %
Apricot stones (AS)	Gr-AS	Aggregates and fragile fragments	~600–90 nm	3.1
Walnut shells (WSh)	Gr-WSh	Aggregates and fragile fragments	~700–85 nm	2.9

**Table 2:** The Raman spectroscopy parameters.

ID Sample	Ratio of Biomass/KOH, g/g	I_G_	I_G_	I_2D_	I_G_/I_2D_	I_D_/I_G_	Number of Layers
Gr-AS	1/5	271	729	471	1.54	0.28	4–5
Gr-WSh	1/5	423	1462	712	2.05	0.37	4–5

**Table 3:** Results of texture characteristics of the studied biomass-derived graphene.

Biomass-Derived Graphene	Specific Surface Area, m^2^/g	Specific Pore Volume, cm^3^/g	Average Pore Size, nm
Gr-AS	1358.680	0.058	25.321
Gr-WSh	1235.491	0.086	25.723

**Table 4:** Comparison of the specific surface area of pre-carbonized materials with similar data from the literature.

Material Type	Carbonation Conditions	Specific Surface Area, m^2^/g	Ref
Biomass-derived graphene	850 °C, 45 min, Ar	350–450	The present study
Coconut husk	700 °C, 1.5 h, N_2_	250–300	[17]
Corn biomass	650 °C, 1 h, Ar	220–310	[18]
Phenol formaldehyde resin	800 °C, 2 h, N_2_	350–400	[19]
Modified cellulose precursor	700 °C, 1 h, CO_2_	280–360	[20,21]

**Table 5:** Comparison of characteristics with previous works.

Sample	Specific Surface Area, m^2^/g	I_G_/I_2D_ Ratio	Carbon Content	Ref
Biomass-derived graphene	~1300	1.61 ± 0.10	75 wt.%	The present study
Rice husk-derived graphene	1001–1556	1.34 ± 0.10	-	[27]
	-	0.9024	-	[28]
Onion husk-derived graphene	1924	1.4 ± 0.10	98 wt.%	[10]

**Table 6:** Technical specifications of high temperature vertical tube furnace (VTF-1200C).

Maximum Heating Temperature	From 40 to 1200 °C
Power consumption	3 × 2 kW = 6 kW (power of one wire: 2 kW, 220 V, R = 24.2 Ohm)
Maximum heating temperature	1200 °C (<1 h)
Camera material	Aluminosilicate ceramics
Dimensions of the working chamber	OD 220 × 1D120 × 720 mm,
Chamber volume	8138 cm^3^
Resistance wire	HRE, diameter 1.5 mm, length 29.48 m.
Temperature controller block	automatic switch, is a short circuit protection; electromagnetic starter—switches heating elements on and off; double START-STOP button—manual heating control; programmable temperature controller—sets and maintains the required (can be specified, defined) temperature; power section on thyristors provides smooth regulation of heating power.
Temperature sensor	Thermocouple type K (chromel-alumel)

**Table 7:** Sequence of stages for obtaining graphene-containing materials from biomass and corresponding processing parameters.

Stage	Conditions and Parameters	Description of the Process
Washing and drying	Repeated washing with distilled water; drying at 383 K, 1 h	Removal of impurities
Pre-carbonation	523–573 K, 45 min, Ar, 5 cm^3^/min, rotating reactor	Initial stage of heat treatment
Desilication	1 M NaOH (60 g in 3 L), 353 K, 3 h	Removal of silicon compounds
Washing and drying after desilication	Wash to neutral pH, dry at 383 K, 2 h	Preparing for activation
Chemical activation	Mixture with KOH (1:5), pressing, annealing in Ar at 1123 K, 2 h	Formation of a porous structure
Washing and drying of activated material	Wash to neutrality, dry at 373 K, 24 h	Removal of reagent residues
Exfoliation	Treatment 37% H_2_O_2_, 60 °C, continuous magnetic stirring at 400 rpm, 48 h, ratio of 1:10 (solid to liquid, *w*/*v*), wash-dry	Removal of amorphous carbon
Product yield	~3% by weight	Final products: Gr-RH, Gr-WSh, Gr-AS

**Table 8:** Parameters of the devices used.

Method of Analysis	Device	Main Parameters	Location of the Event
SEM	Nova NanoSem 450 FEI/Termofisher (Waltham, MA, USA)	at 3.00 kV in high vacuum mode, using an Everhart Thornley Detector (ETD) and Through the Lens Detector (TLD) for details micrographs and elemental microanalysis (EDX) at 15.00 kV	“ACE” laboratory of University of Naples Federico II (Naples, Italy)
Elemental analysis	PRIMACS100 analyzer (Skalar Analytical B.V., Breda, The Netherlands) and CHN 628 LECO elemental analyzer (LECO Corporation, St. Joseph, MI, USA)	ASTM E870 procedure using EDTA as standard	“ACE” laboratory of University of Naples Federico II (Naples, Italy)
Raman spectroscopy	Jasco NRS-3100 (Jasco, Tokyo, Japan)	Laser 514 nm, spectral resolution 4 cm^−1^, accumulation time 30 s	National Open Nanotechnology Laboratory (Almaty, Kazakhstan)
Specific surface area analysis (BET method)	Sorbtometer-M (Thermo Fisher Scientific, Waltham, MA, USA)	Low temperature nitrogen adsorption, surface area range: 1–3000 m^2^/g	Institute of Combustion Problems (Almaty, Kazakhstan)
Transmission electron microscopy	HRTEM, FEI Talos F200X G2, USA (Thermo Fisher Scientific, Waltham, MA, USA)	at accelerating voltages between 200 kV and 300 kV, with the powder specimens dispersed onto copper grids coated with ultrathin carbon films	State Key Laboratory of Precision Blasting, Jianghan University (Wuhan, China)

## Data Availability

The original contributions presented in this study are included in the article. Further inquiries can be directed to the corresponding authors.

## Abbreviations

The following abbreviations are used in this manuscript:

RH	Rise husk
AS	Apricot stones
WSh	Walnut shells
SEM	Scanning electron microscope
BET	Brunauer–Emmett–Teller method
CVD	Chemical vapor deposition
